# Navigating Complexity: A Case Report of Concurrent Central Cord Syndrome and Stroke in an Elderly Gentleman

**DOI:** 10.7759/cureus.51789

**Published:** 2024-01-07

**Authors:** Hoe Teong Kee, Mohd Hezery Bin Harun, Fadzrul Abbas Mohamed Ramlee, Teck Siang Lim, Fahrudin Che-Hamzah

**Affiliations:** 1 Orthopaedics and Traumatology, Hospital Sultan Abdul Aziz Shah, University of Putra Malaysia, Serdang, MYS; 2 Orthopaedic Surgery, Hospital Sultan Abdul Aziz Shah, University of Putra Malaysia, Serdang, MYS

**Keywords:** spinal decompression, neuro-imaging, cerebro-vascular accident (stroke), cervical spinal cord injury, central cord syndrome

## Abstract

Central cord syndrome (CCS) predominantly manifests in elderly individuals with pre-existing cervical spondylosis resulting from hyperextension mechanisms. However, it is not exclusive to the older population and can occur in younger individuals following traumatic cervical spine injuries or, less frequently, due to nontraumatic causes. The impact of this syndrome is more pronounced in the upper extremities, where motor function experiences greater impairment compared to sensory function. CCS presents itself along a spectrum of severity. At one end, individuals may exhibit weakness confined to the hands and forearms while preserving sensory function. At the other extreme, complete quadriparesis may occur, albeit with sacral sparing being the sole indication of an incomplete spinal cord injury. This spectrum underscores the varied and nuanced clinical presentations within CCS. Moreover, concurrent acute stroke presentations can mimic CCS symptoms, further complicating the diagnostic process. The challenge lies in differentiating these two distinct conditions, particularly in an elderly population with overlapping risk factors. This diagnostic challenge adds a layer of complexity to clinical decision-making and underscores the importance of comprehensive evaluations in patients presenting with neurological symptoms. This case report presents a 73-year-old gentleman with a history of a recent stroke and motor vehicle accidents, highlighting the diagnostic challenges and multidisciplinary management required for concurrent CCS and stroke mimicry. This report is unique, as there are no existing case report publications detailing concurrent CCS and stroke. It emphasizes the necessity for a comprehensive diagnostic approach and coordinated care in managing such intricate cases.

## Introduction

Central cord syndrome (CCS) is a subtype of incomplete spinal cord injury characterized by motor and sensory deficits predominantly affecting the upper extremities, a consequence often observed after hyperextension injuries to the cervical spine [1]. The incidence of acute traumatic central cord syndrome (ATCCS) exhibits a bimodal distribution, impacting individuals under the age of 30 years following high-impact trauma and affecting elderly patients after low-energy injuries [2]. The etiology of ATCCS is primarily linked to hyperextension injuries of the cervical spine. Typically, the spinal cord sustains injury due to an anterior-posterior compression force, particularly in individuals with pre-existing cervical stenosis and spondylosis, which predisposes them to cord injury [3]. This classic mechanism involves a compression force affecting the spinal cord, even in the absence of evident bony injury [4]. Moreover, ATCCS can also manifest after cervical spine fractures, whether with or without dislocation, and as a consequence of acute disc herniation. This multifaceted presentation underscores the diverse ways in which the spinal cord can be compromised in traumatic scenarios. Anteriorly, the presence of osteophytes, as well as calcified or noncalcified herniated discs, may contribute to the narrowing of the spinal canal at specific focal areas [5]. While CCS is frequently associated with traumatic incidents, the co-occurrence with a recent stroke introduces a level of rarity that demands attention and exploration. The interplay between spinal and cerebral pathologies in this patient poses a diagnostic conundrum, emphasizing the necessity for healthcare professionals to expand their considerations beyond typical presentations. This introduction aims to set the stage for the intricacy and clinical significance of the case, prompting a deeper exploration into the diagnostic challenges posed by the simultaneous occurrence of CCS and stroke. This atypical manifestation adds layers of complexity to the diagnostic process, challenging conventional expectations and underscoring the need for a nuanced understanding of this condition.

## Case presentation

The patient, a 73-year-old active smoker with a 30-pack-year history of smoking, hypertension, and dyslipidemia, presented with a sudden onset of right-sided lower limb weakness and numbness over the past day. Notably, he reported experiencing weakness in both hands for the preceding week, characterized by a weak finger grip, clumsiness, and difficulties with basic tasks such as buttoning clothes and combing hair.

Upon further inquiry, the patient disclosed a recent motor vehicle accident, occurring a week ago, during which he skidded on his motorbike, resulting in a fall towards his left side with abrupt flexion-extension of his neck. Interestingly, post-trauma, he did not initially experience any limb weakness and maintained the ability to ambulate without aid.

During the neurological examination, significant findings included muscle strength measured as a power of 0/5 over the right lower limb from L2 to S1, 5/5 over the right upper limb from C5 to C6, and 0/5 over C7 to T1. In contrast, the left upper and lower limbs exhibited full power at 5/5. The patient demonstrated hypertonia in both upper and lower limbs, and reflexes such as biceps, triceps, and reverse brachioradialis were hyperreflexic. The Hoffmann test was positive, indicating an upper motor neuron lesion. Importantly, there was no cervical tenderness, and the neck’s range of motion was full. The patient exhibited slurred speech and facial drooping. Blood pressure monitoring revealed persistently elevated systolic pressure ranging from 140 to 180 mmHg and diastolic blood pressure between 100 and 120 mmHg. The fasting lipid profile showed total cholesterol at 6.2 mmol/L, low-density lipoprotein at 4.1 mmol/L, high-density lipoprotein at 1.0 mmol/L, and triglycerides at 2.8 mmol/L. For this patient, a Recognition of Stroke in the Emergency Room (ROSIER) Scale stroke assessment score of +4 suggests a likely diagnosis of acute stroke.

**Figure 1:**
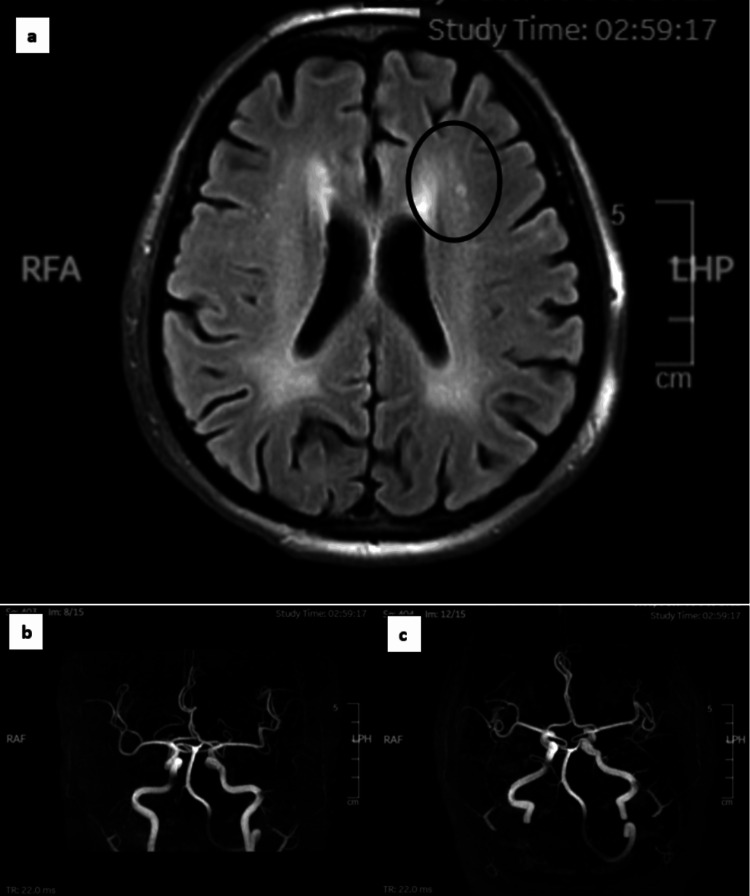
Neuro-imaging in the acute setting. a) MRI of the brain (axial DWI) reveals a small (<15mm in diameter) and deep left lacunar infarction (depicted by the black circle). b) Magnetic resonance angiography (MRA) of the Circle of Willis (anteroposterior view) demonstrates the continuous structure of the Circle of Willis, with no stenosis observed along the basilar artery, middle cerebral arteries, and penetrating arteries. c) Magnetic resonance angiography (MRA) of the Circle of Willis (infero-superior view) provides a different perspective, confirming the continuity of the Circle of Willis, with no evident stenosis changes observed along the basilar artery, middle cerebral arteries, and penetrating arteries.

Initially, CT and magnetic resonance angiography (MRA) of the brain revealed a left lacunar infarct, leading to the provisional diagnosis of a recent left lacunar infarct with right hemiparesis (Figure 1a-1c). Identified risk factors included chronic heavy smoking, chronic uncontrolled hypertension, and dyslipidemia. Antiplatelet medication, specifically aspirin 300 mg daily, was initiated, along with blood pressure optimization through medication, smoking cessation, and the commencement of post-stroke rehabilitation. However, the patient's neurological symptoms deteriorated significantly over the next two days. He experienced motor weakness in both upper and lower limbs. Upon re-examination, the bilateral upper limb power was 5/5 over C5 to C6 and 0/5 over C7 to T1, while sensation was 2/2 over C5 to C6 and 0/2 over C7 to T1. Remarkably, the right lower limb power remained unchanged at 0/5 from L2 to S1, but the left lower limb power was now reduced to 3/5 from L2 to S1. The anal tone was intact, the perianal sensation was intact, and the bulbocavernosus reflex (BCR) was present.

**Figure 2:**
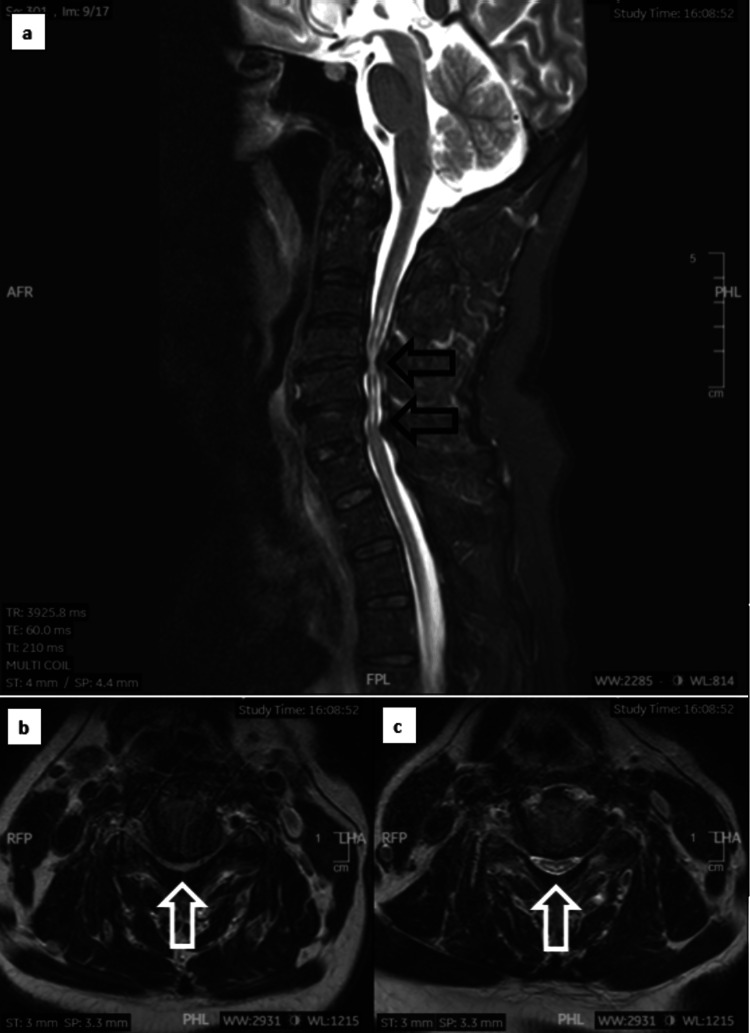
MRI of the cervical spine in various views. a) Spinal cord compression with cord changes spanning from C4 to C6, with a more severe level of compression observed at the C4/C5 level (indicated by the black arrow) (sagittal view). b) Severe central cervical canal stenosis at the C4/C5 level, characterized by complete cerebrospinal fluid effacement and evident cord compression (indicated by the white arrow) (axial view). c) Moderate central cervical canal stenosis with near-complete cerebrospinal fluid effacement and cord indentation at the C5/C6 level (indicated by the white arrow) (axial view).

**Figure 3:**
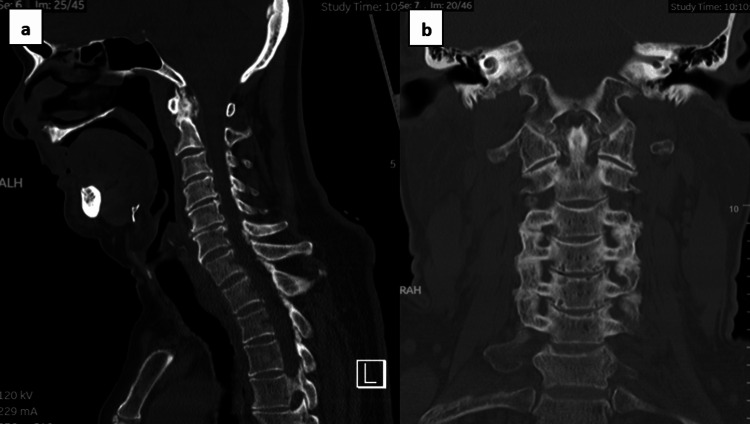
Computed tomography of the cervical spine. a) Sagittal view computed tomography of the cervical spine revealing osteophyte formation over the cervical vertebrae, with no evidence of ossification of the posterior longitudinal ligament. b) Coronal view computed tomography of the cervical spine showing uncovertebral hypertrophy without any observed vertebral fractures, subluxations, or dislocations.

An emergency MRI of the cervical spine was conducted, revealing spinal cord compression with cord changes from C4 to C6, most pronounced at the C4/C5 level, and absolute spinal canal stenosis. These findings led to the diagnosis of CCS, which encompassed intervertebral disc protrusion, hypertrophy of the ligamentum flavum, and the formation of osteophytes (Figure 2a-2c). An emergency CT scan of the cervical spine was also performed to assess the presence of ossification of the posterior longitudinal ligament and the formation of bony osteophytes. This examination aimed to identify the source of spinal canal compression and to facilitate appropriate pre-operative planning (Figure 3a-3b). These findings indicated an urgent and critical condition, necessitating immediate surgical decompression and stabilization of the cervical spine. This unexpected turn of events challenged the initial diagnosis, prompting a reevaluation of both concurrent spinal and cerebral pathologies. The intricate interplay between the recent traumatic incident and the evolving neurological symptoms underscored the need for a comprehensive diagnostic approach to unravel the complexities of this case.

**Figure 4:**
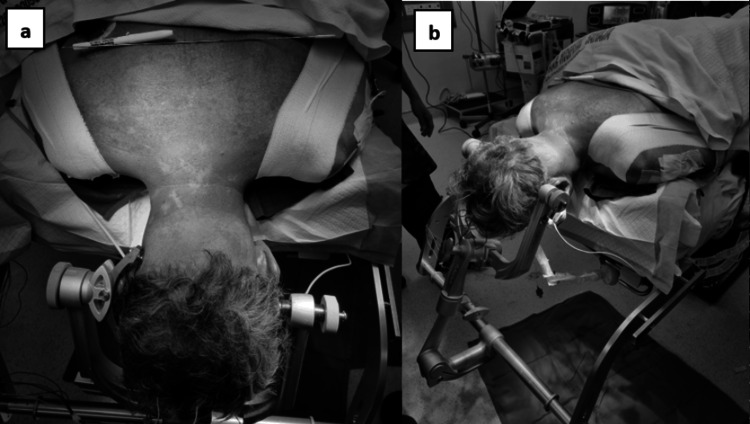
Clinical pictures of intra-operative positioning. The clinical picture shows the patient lying prone, with the head securely held in place using a Mayfield® Cranial Stabilization System, with pin placement over the sweatband zone. Additionally, the bilateral shoulders are pulled and strapped with an adhesive strap to tense the skin over the operative field. a) Superior view, b) Side view.

In response to evolving clinical examinations and the diagnosis of concurrent traumatic central cord syndrome and acute left lacunar infarct, significant compression of the cervical spinal cord was identified as leading to a rapid progression of neurological deficits. Consequently, the decision was made to opt for direct posterior decompression of the spinal canal. This involved performing a laminectomy from C4 to C6, coupled with stabilization using posterior instrumentation and fusion from C4 to C6. The rationale for choosing the posterior approach included the absence of ossification of the posterior longitudinal ligament (OPLL), ligamentum flavum hypertrophy, disc height restoration through rod distraction, and the opportunity for direct visualization of the dura mater following laminectomy. The patient provided consent for the operation and underwent optimization under a multidisciplinary team. The emergency operation took place the following day. In this surgical procedure, the patient, positioned in a prone stance with head stabilization via a Mayfield clamp, underwent general anesthesia (Figure 4a-4b). Intravenous prophylactic antibiotic Rocephin 2g was administered prior to induction. The surgical team meticulously prepared the operative area, initiating a midline incision over the C4 to C7 spinous process level. Subcutaneous tissue was sequentially opened in layers, allowing for subperiosteal elevation of paraspinal muscles.

**Figure 5:**
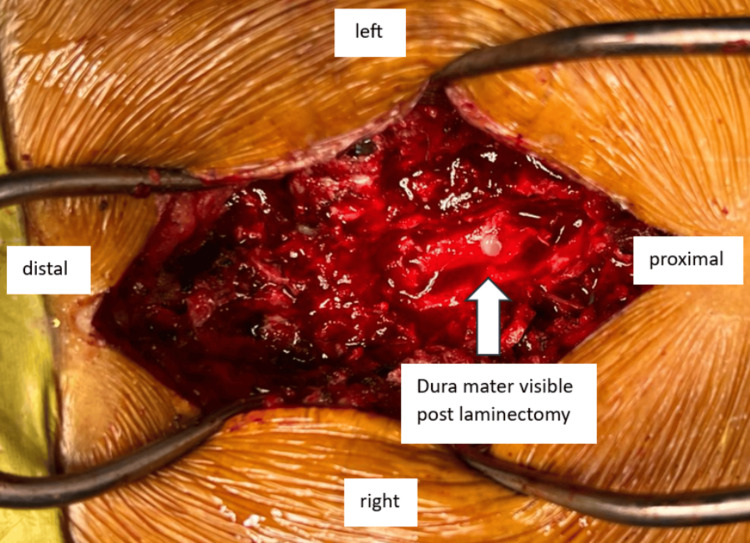
The intraoperative clinical picture demonstrates successful decompression of the spinal cord following laminectomy from C4 to C6, with an intact dura mater and no evidence of cerebrospinal fluid leakage.

The surgical findings revealed a series of critical observations. Firstly, surgical exploration uncovered spinal cord compression extending from C4 to C6, with a notable absence of vertebral fractures. The posterior ligamentous complex was intact, and the presence of calcified ligamentum nuchae was noted. Spinous processes from C4 to C6 were removed, and laminectomy was performed using a diamond burr and Kerrison rongeur under loupe magnification. Following the laminectomy, hypertrophied ligamentum flavum was excised with Kerrison rongeur until bulging of the spinal cord was observed. Fortunately, the dura mater remained intact with no observed cerebrospinal fluid (CSF) leakage (Figure 5).

**Figure 6:**
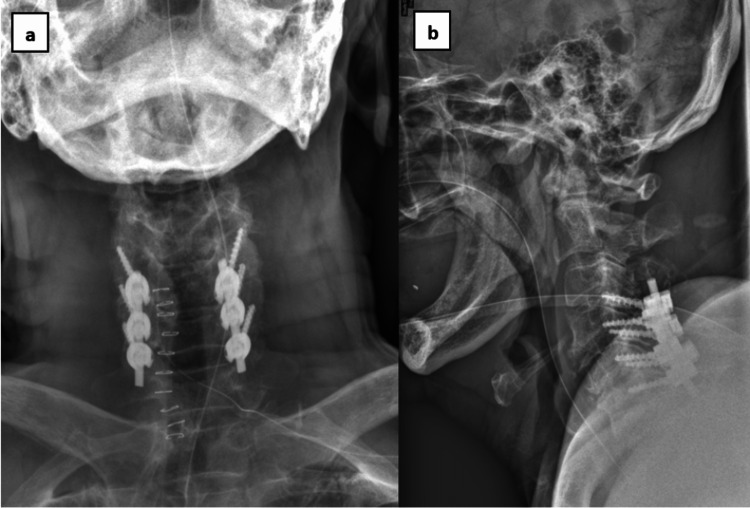
Post-operative cervical radiographs reveal the accurate trajectory of lateral mass screws, along with the restoration of cervical lordosis and disc height. a) anteroposterior view, b) lateral view

The implants utilized during the procedure, sourced from NuVasive®, United States, consisted of six 3.5 mm lateral mass screws, each measuring 16 mm in length, and two rods securely fixed with screw nuts from C4 to C6. Intraoperative neuromonitoring showed a positive trajectory, with improvement in the signal from C3 to C8 compared to the post-operation baseline. The operative steps involved meticulous identification of screw entry points, burring of the cortex, and subsequent insertion of lateral mass screws using the Magerl technique, all guided by intraoperative imaging. Posterolateral fusion was achieved by inserting an autologous bone graft harvested from the spinous process and synthetic bone graft granules. The final reduction was deemed acceptable under intraoperative imaging guidance (Figure 6a-6b).

Wound closure involved a comprehensive approach, including closure of the fascia with Vicryl 1®, Ethicon Inc., insertion of Vancomycin powder over the subcutaneous tissue, closure of the subcutaneous tissue with Vicryl 2/0®, Ethicon Inc., and skin approximation using a stapler. Sterile dressings were then applied to complete the surgical intervention.

**Figure 7:**
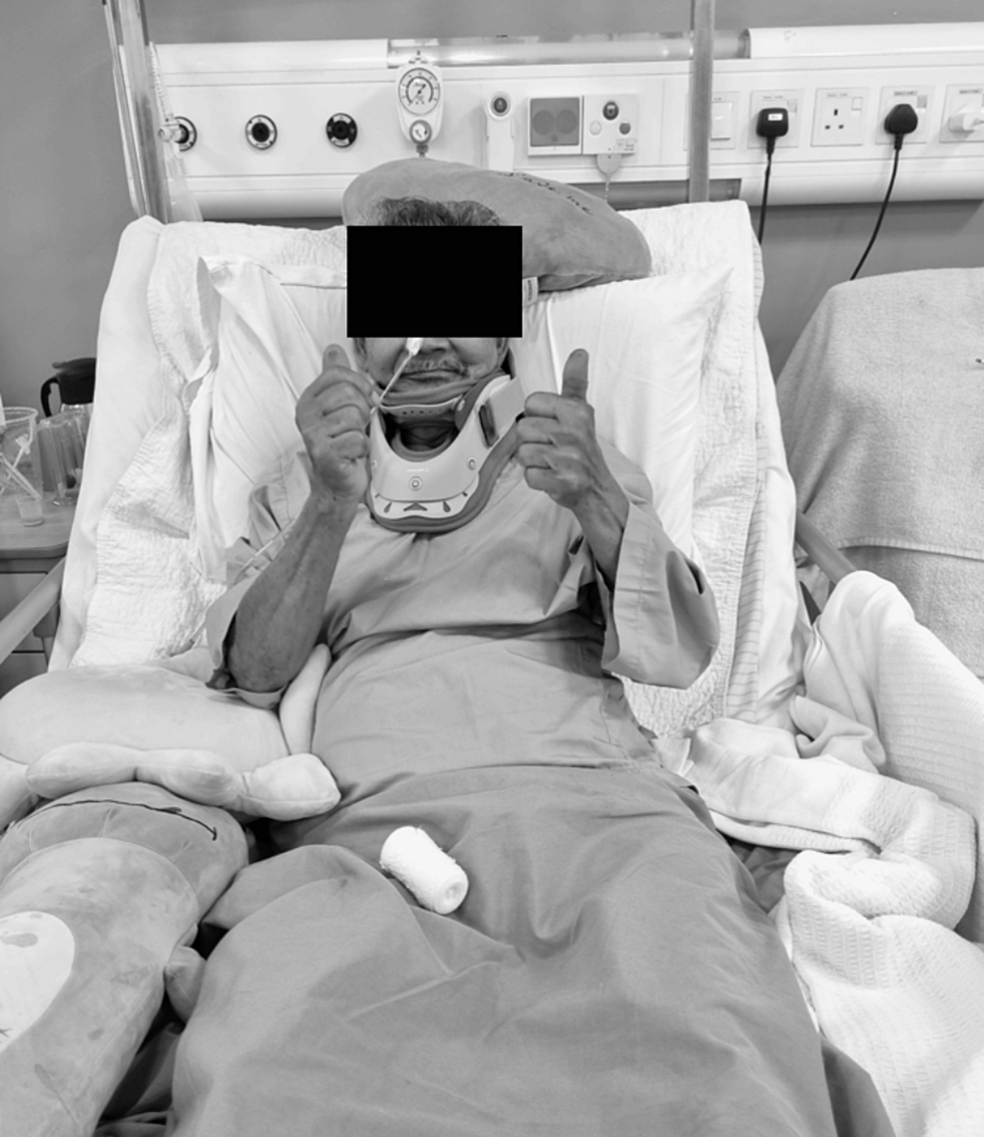
The postoperative clinical picture indicates a favorable prognosis, demonstrating the recovery of bilateral upper limb function with the ability to perform a 'thumbs-up' gesture, signifying the restoration of motor power from C7 to T1.

Postoperatively, an Aspen collar was applied. The patient tolerated the three-hour operation well, requiring a pint of packed RBC transfusion, and was subsequently admitted to the ICU for postoperative monitoring. Two days after the operation, he was discharged to the general ward in good condition. He was then referred to inpatient physiotherapy for limb and chest physiotherapy, intensive spirometry, wheelchair ambulation, and the prevention of complications associated with prolonged immobilization. Intravenous antibiotic Rocephin 1g twice a day was administered for three days. The patient demonstrated significant improvement in his neurological assessment and expressed motivation to reintegrate into society. One week post-operation, he was discharged, demonstrating the ability to perform bed and wheelchair transfers, stand with assistance, and exhibit good upper limb function (Figure 7).

This marked improvement in functional abilities indicates a positive response to the surgical intervention. The patient's successful performance of various activities of daily living reflects both the effectiveness of the treatment and the individual's commitment to rehabilitation. The multidisciplinary approach, which included physiotherapy and comprehensive post-operative care, likely played a crucial role in achieving these positive outcomes. Ongoing support and monitoring will be essential to ensure the patient's continued progress and successful reintegration into daily life. Upon discharge, the patient was prescribed the following medications: Cardiprin 100 mg daily, Rosuvastatin 20 mg daily, Calcium Carbonate 500 mg twice a day, and Rocaltrol 0.25 mcg daily.

## Discussion

The intricacies of differentiating symptoms between CCS and stroke present a diagnostic challenge, particularly in high-risk individuals with overlapping neurological presentations. 

Challenges in differential diagnosis

The diagnostic journey is fraught with challenges in distinguishing symptoms arising from CCS and stroke. In the elderly, the presence of acute stroke introduces complexity, as symptoms may mimic those of spinal pathology, creating a diagnostic dilemma. The clinical manifestations, including limb weakness, sensory deficits, and impaired motor function, may overlap, necessitating a thorough evaluation to discern the underlying pathology accurately. Moreover, studies have indicated that elderly individuals with substantial comorbidities experience a less favorable prognosis, a diminished likelihood of undergoing operative intervention, and a higher inpatient mortality rate [6]. In a distinct investigation, Schroeder GD et al. discovered that patients exhibiting elevated T2 signal intensity in their spinal cord on MRI demonstrated a more severe initial neurological injury but exhibited minimal early deterioration compared to those with lower cord signal intensity [7]. The discrepancy between clinical presentation and imaging results emphasizes the need for meticulous investigation, raising questions about the relationship between the recent motor vehicle accident, the stroke, and the concurrent spinal pathology.

Rationale behind surgical intervention

The decision for an emergent surgical intervention involving laminectomy from C4 to C6 with posterior instrumentation stems from the imperative to address the cord compression noted in the MRI findings. Uribe J et al. [8] presented findings from a retrospective case series involving patients treated for ATCCS without fracture or dislocation through expansile laminoplasty. At the three-month follow-up, 71% of individuals exhibited an improvement of one ASIA Impairment Scale (AIS) grade. In cases of individuals diagnosed with ATCCS and persistent spinal cord compression, Chen TY et al. demonstrated enhanced neurological recovery through surgical decompression. The study also revealed a reduced length of hospital stay and a diminished likelihood of developing chronic myelopathy [9]. La Rosa G et al. [10] conducted a systematic review assessing the timing of surgical decompression following spinal cord injury (SCI). Their findings indicated that individuals with acute SCI who underwent early surgical intervention exhibited better outcomes compared to those who received delayed surgical intervention or conservative treatment. The 'Surgical Timing in Acute Spinal Cord Injury Study' specifically investigated the impact of early surgical intervention (defined as within 24 hours of injury) versus delayed surgery (performed more than 24 hours after injury) on patients with acute SCI.

Holistic and multidisciplinary approach

The discussion underscores the paramount importance of adopting a holistic and multidisciplinary approach in managing concurrent CCS and stroke. The collaboration between neurologists, orthopedic surgeons, and imaging specialists is highlighted as crucial for accurate diagnosis and optimal treatment planning [11]. The integration of neuro-monitoring during surgery adds a layer of precision, contributing to improved postoperative outcomes.

Optimizing patient outcomes

Highlighting the importance of a thorough diagnostic approach, the discussion emphasizes the necessity for continuous postoperative care, encompassing rehabilitation and physiotherapy. The primary emphasis in physical therapy for CCS lies in preserving range of motion (ROM) and improving mobility skills [12]. It is crucial to strengthen any remaining lower extremity musculature, enhance trunk balance, and provide stabilization. Achieving safe transfers and wheelchair mobility stands as an additional objective that should be met before commencing gait training. The observed successful recovery, marked by the restoration of upper limb function, affirms the efficacy of the selected intervention. This underscores the significance of a patient-centric, multidisciplinary approach to maximizing outcomes. In summary, this discussion navigates the complexities of concurrent CCS and stroke, shedding light on the challenges in diagnosis, unexpected imaging findings, the rationale behind surgical intervention, and the pivotal role of a holistic and multidisciplinary approach in optimizing patient outcomes. The presented case serves as a valuable contribution to the understanding of intricate neurological presentations in high-risk individuals, offering insights that can guide future clinical approaches and research endeavors.

## Conclusions

Concurrent CCS and stroke present diagnostic and therapeutic dilemmas. This case report illustrates the complexities involved in navigating such scenarios and highlights the critical role of comprehensive evaluation, timely neuroimaging, and coordinated surgical intervention. The positive outcomes for our patients underscore the importance of a multidisciplinary response in optimizing patient care.
